# Pharmacologic modulation of experimentally induced allergic asthma

**DOI:** 10.2478/v10102-011-0006-x

**Published:** 2011-03

**Authors:** Soňa Fraňová, Anna Strapková, Juraj Mokrý, Martina Šutovská, Marta Jošková, Vladimíra Sadloňová, Martina Antošová, Darina Pavelčíková, Dana Flešková, Gabriela Nosáĺová

**Affiliations:** Department of Pharmacology, Jessenius Faculty of Medicine, Comenius University, Martin, Slovakia

**Keywords:** allergic asthma, nitric oxide, ion channels, PDE, polyphenols

## Abstract

Allergic asthma is the most frequent disease of the respiratory tract. The aim of the current experimental and clinical studies was to find new sources of drugs able to control asthmatic inflammation and airway hyperresponsiveness. Our experimental studies were focused on efficiency evaluation of substances able to influence activities of ion channels, phosphodiesterase (PDE) isoforms, substances from the group of polyphenols and NO metabolism modulators during experimentally induced allergic asthma.

## Introduction

Allergic asthma is a chronic inflammatory disease of the airways. Characteristic features of allergic asthma are allergen-induced early and late bronchial obstructive reactions, airway inflammation, structural changes of the airway wall associated with progressive decline in lung function, and airway hyperresponsiveness (AHR). AHR is defined by an exaggerated obstructive response of the airways to a variety of pharmacological, chemical and physical stimuli (Meurs *et al*., 2008)

In addition to smooth muscle hyperreactivity, allergic asthma is associated with ongoing airway inflammation, infiltration of the airway wall by inflammatory cells (eosinophils, lymphocytes, especially CD4-positive T-cells) and formation of Th2 cytokines (IL-4, IL-5, IL-13), which play a key role in orchestrating the eosinophilic inflammatory response (Antoniu, 2009). Inflammation is thought to cause symptoms of asthma directly and indirectly by inducing contraction of airway smooth muscle, enhancing airway responsiveness to various stimuli and by inducing changes in structural components of the airway wall leading to airway remodelling (Zuyderduyn *et al*., 2008).

The aim of our experimental studies related to allergic asthma was to find new sources of drugs able to modulate smooth muscle hyperreactivity and the degree of inflammation of the airways. Different experimental models of airways hyperreactivity were used for experimental simulation and of allergic asthma. We utilized the model of guinea pig airways hyperreactivity induced by allergen – ovalbumin (OVA) parenteral administration and inhalation.

## Allergic asthma and nitric oxide metabolism

The precise pathomechanism of airway hyperreactivity is unknown but many studies assume that nitric oxide (NO) plays a very important role in this process (Ricciardolo, 2003). We studied the effects of the modulation of NO level on the airways smooth muscle response in conditions of experimental hyperreactivity at three levels: the level of the precursor changes (L-arginine supplementation or treatment with drugs releasing this molecule – NO donors); activity of enzymes involved in NO homeostasis (NO synthases – NOS, arginases); and mechanism of NO action (guanylylcyclase). The changes in NO metabolism during allergic inflammation were compared with pathological NO changes induced by a chemical trigger – exogenous irritant, *i.e.* toluene vapor.

For the changes of NO production, we used inhibition of NOS activity by non-specific (N^ω^-nitro-L-arginine methyl ester – L-NAME) or specific inhibitor (aminoguanidine – AG). They were applied *in vivo* during two therapeutic regimens (acute or chronic) in toluene- or allergen-induced hyperreactivity in guinea pigs. It is interesting that we recorded a significant decrease of tracheal smooth muscle reactivity after acute L-NAME pre-treatment in toluene-induced hyperreactivity but an opposite effect, increase of tracheal smooth muscle reactivity, in allergen-induced hyperreactivity after acute and chronic L-NAME pre-treatment. The lung tissue reactivity was reduced after acute and chronic L-NAME pre-treatment in toluene-induced hyperreactivity but changes in allergen-induced hyperreactivity were non-significant. Our study demonstrated that the effect of NOS inhibitors on the airway reactivity changes was dependent on the hyperreactivity provoking factor and type of therapeutic regimen. Constitutive isoforms of NOS have probably a more significant role in allergen-induced airways hyperreactivity than in toluene-induced hyperreactivity (Antošová *et al*., 2008).

The limitation of L-arginine bioavailability by arginase for NO synthesis may contribute to airway hyperreactivity. We investigated the effect of changes in arginase activity in ovalbumin-induced airway hyperreactivity after *in vitro* administration of arginase or non-selective inhibitor of arginase N^ω^-hydroxy-L-arginine (NOHA) (Antošová *et al*., 2006). We did not record significant differences in the reactivity of tracheal and lung tissue smooth muscle if we applied arginase *in vitro*. NOHA *in vitro* induced a dose-dependent decrease of tracheal and lung tissue smooth muscle reactivity (Strapková and Antošová, 2009). The supplemention of L-arginine together with NOHA *in vitro* intensified the decrease of the airways reactivity induced by an inhibition of arginase, confirming the importance of the optimal level of L-arginine for control of bronchomotor tone.

Since NOS and arginase compete for the common substrate L-arginine, we analysed the response of tracheal and lung tissue smooth muscle after administration of inhibitors of NOS – (L-NAME and AG) and the inhibitor of arginase (NOHA) in combinations under *in vitro* conditions. We found a decrease of ovalbumin-induced hyperreactivity if we used the combination of NOS and arginase inhibitors. Simultaneous inhibition of iNOS (AG) and arginase (NOHA) evoked the most expressive effect. Inhibition of both enzymes caused a more expressive effect in tracheal smooth muscles than in the lung. The results point out the importance of an optimal balance in the activity of NO synthases and arginase. They confirmed that competition of NO synthases and arginase for the common substrate L-arginine may be one of the important factors influencing the condition of the bronchomotoric tone in the airway hyperreactivity (Strapková *et al*., 2008, Strapková and Antošová, 2011).

## Allergic asthma and modulated activity of ion channels

Allergic inflammatory disease of the airways is a sophisticated process, mostly characterized by representative histopathological features associated with typical clinical symptoms. Development of allergic airways inflammation is closely associated with modulated activity of calcium and potassium ion channels resulting in production of antigen-specific antibodies, qualitatively changed mucus output, mast cell differentiation and airways eosinophilia (Pelaia *et al*., 2002). Thus, ion channels activity is finally responsible for clinical manifestations as obstruction, hyperreactivity and airways remodeling, as well as pathological cough. Modern pharmacotherapy of bronchial asthma is based on regularly administered disease controllers (*e.g.* inhaled corticosteroids, long-acting β_2_-adrenomimetics (LABA), selective inhibitors of PDE, etc.) and actually applied relievers (*e.g.* short-acting β_2_-adrenomimetics (SABA) or anticholinergics) (Beckett *et al*., 2003). All these drugs from miscellaneous pharmacological groups have one common attribute: their mechanism of action is partly mediated via changed activity of certain ion channels (calcium, potassium or chloride) type. Recent knowledge concerning asthma pathophysiology and the role of ion channels has opened the possibility of finding tissue-selective ion channel modulators as a novel target for asthma therapy.

## Potassium ion channels

**Figure 1:**
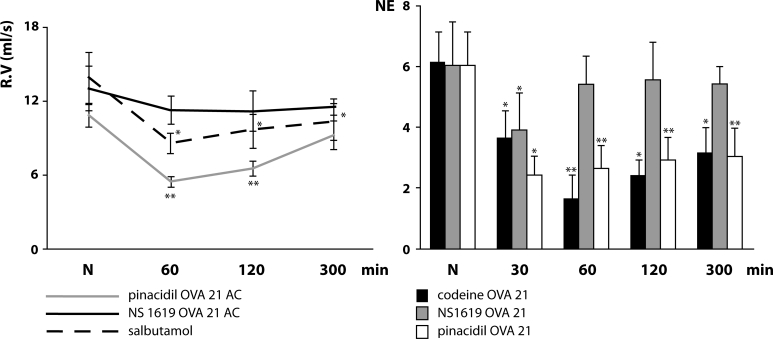
Changes of specific airway resistance (left side – R.V) and number of cough efforts (right side – NE) on administration of K^+^_ATP_ agonist (pinacidil) and BK^+^_Ca_ agonist (NS 1619 OVA 21 AC) measured in guinea pigs sensitized by ovalbumin (on 21^st^ day of sensitization) and compared with control drugs (R.V – salbutamol and NE – codeine). *p<0.05; **p<0.01.

Previously, it was shown that activation of K^+^_ATP_ as well as BK^+^_Ca_ ion channels in airways of unsensitised guinea pigs resulted in significant suppression of cough reflex and bronchoconstriction experimentally induced by citric acid aerosol (Sutovska *et al*., 2007). Recently we tested the influence of developing allergic inflammatory reaction on the relationship between defense reflexes of the airways and the activity of both potassium ion channel types in guinea pigs sensitised by ovalbumin. The influence on cough reflex was assessed using the method of citric acid induced cough. The airways smooth muscle reactivity *in vivo* was evaluated by actual values of specific airways resistance (RV) according to Pennock *et al*. (1979). It was found that the defense role of K^+^_ATP_ remained almost unchanged even in conditions of allergic inflammation, while the role of BK^+^_Ca_ decreased progressively according to the degree of allergic inflammation (Sutovska *et al*., 2009) (Figure 1). A further important fact was observed after single applied doses: agonists of both potassium ion channels significantly increased an infiltration of pulmonary tissue by lymphocytes and augmented pulmonary eosinophilia. All these findings corresponded with further literature data (Duffy *et al*., 2001) and pointing out the involvement of potassium ion channels in the development of allergic inflammation.

## Calcium ion channels

**Figure 2:**
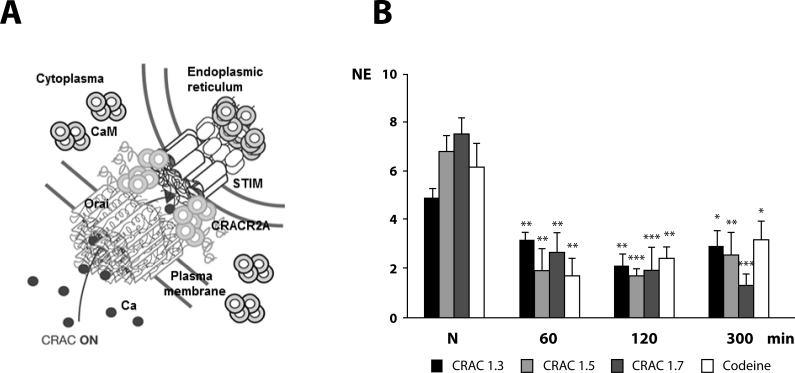
**A** – CRAC ion channel. **B –** Changes in the number of cough efforts (NE) on administration of CRAC ion channels antagonist (3-fluoropyridine-4-carboxylic acid) in different doses measured in guinea pigs sensitized by ovalbumin (on 21^st^ day of sensitization) and compared with codeine. N – before drug administration. *p<0.05; **p<0.01; ***p<0.001.

Highly Ca^2+^-selective, store-operated (CRAC) ion channels (calcium-activated calcium ion channels) represent one of the TRP superfamily channels (transient receptor potential), so-called store-operated channels (SOCs), regulating intracellular transport and metabolism of calcium ions and playing a key role in most cellular processes (Frischauf *et al*., 2008). Detailed examination of the function of CRAC channels located in mast cells and lymphocytes showed association with massive production of proinflammatory mediators. Previously, Peel *et al*. (2008) showed expression and role of CRAC ion channels in contracting activity of human airways smooth muscle. Our experimental studies revealed that the CRAC ion channel antagonist 3-fluoropyridine-4-carboxylic acid (FCA) was an antitussive and bronchodilatory active agent. Intraperitoneally administered FCA in doses of 1.3, 1.5 and 1.7 mg.kg^−1^ caused statistically significant and dose-dependent decrease of NE (number of cough efforts) in conscious OVA-sensitized guinea pigs, which is comparable to the suppressive effect of the centrally acting antitussive agent codeine tested in the control group (dose 10 mg.kg^−1^ i.p., Figure 2). The values of specific airways resistance (RV) were significantly suppressed, similarly as antitussive activity, in a dose-dependent manner, regardless of the bronchoconstrictor used (citric acid c=0.3 M or histamine c=10^−6^ M/l). Besides, the highest experimental dose of the CRAC antagonist exerted a bronchodilatory effect which exceeded the activity of the control drug-salbutamol.

It can be summarized that the presented experiments confirmed the specific role of plasmalemmal potassium and intracellular calcium ion channels in the pathophysiology of disorders accompanied by allergic airways inflammation. K^+^_ATP_ ion channels retained their defense property despite the developing allergic airways inflammation, while the role of BK^+^_Ca_ progressively changed. Accordingly, K^+^_ATP_ represents a more rational therapeutic target for novel antiasthmatic drugs. However, introduction of K^+^_ATP_ agonists in clinical practice is strongly limited by their proinflammatory effects. Agents antagonizing the function of CRAC calcium channels appear to be promising. They may represent a new antiasthmatic therapeutic trend due to their antitussive and bronchodilatory effects and the influence of immune cell-key pathomechanisms of allergic airways inflammation.

## Hyperreactivity of airways and phosphodiesterase (PDE) inhibitors

At our department we have been focused for several years on a pharmacological modulation of the smooth muscle activity in the airways and of the cough reflex by various agents affecting the activity of PDE, *i.e.* xanthine derivatives and PDE inhibitors.

In the therapy of airway diseases associated with cough and inflammation, such as bronchial asthma and chronic obstructive pulmonary disease (COPD), several xanthine derivatives are still used. They are generally considered to be non-selective inhibitors of PDE. Nevertheless, in therapeutically relevant plasma concentrations several other mechanisms are involved in their effects, *e.g.* antagonism of adenosine receptors, activation of histone-deacetylases and others (for review see Mokra and Mokry, 2007). Furthermore, low specificity of their mechanism of action, interactions with other drugs, and a narrow therapeutic range can often lead to occurrence of adverse effects, which can limit their use as antitussives (Antoniu, 2006). Therefore on using selective or dual PDE inhibitors in the therapy of these diseases and in affecting cough their effect should be better understood.

Bronchodilation and an anti-inflammatory action of non-selective PDE inhibitors has been only partially elucidated and little is known about the antitussive effects of xanthine derivatives (Mokry *et al*., 2008) or selective PDE inhibitors (PDE3, PDE4, PDE5) (Fujimura and Liu, 2007). Selective inhibitors of PDE have attracted increasing attention in the therapy of respiratory diseases (Chung, 2006). PDE isoenzymes play an important role in the regulation of airways diameter and smooth muscle functions. PDE3 and PDE4, both hydrolyzing cAMP, were confirmed as major PDE isoforms in the airways. However, airway smooth muscle contains more PDE isoenzymes, *e.g.* PDE1,3,4,5 and 7.

The antitussive properties of xanthine derivatives theophylline and theobromine as non-selective PDE inhibitors on citric acid induced cough were studied. Furthermore, the participation of PDE1, PDE3, PDE4, and PDE5 isoenzymes in cough and antitussive effects of their selective inhibitors was assessed at our department.

The administration of some xanthine derivatives (aminophylline, caffeine, and theophylline) led to a significant decrease of specific airway resistance, associated with changes in minute ventilation caused predominantly by influencing the frequency of breathing. The previously demonstrated antitussive effect of intraperitoneally administered theophylline to conscious cats after mechanical stimulation of the laryng was confirmed also in amodel of chemically induced cough in guinea pigs. The antitussive activity of theophylline was even higher compared to the commercially used non-narcotic antitussive dextromethorphan as well as other agents of plant origin, *e.g.* *Emblica officinalis*, *Paederia foetida,* etc. (Nosalova *et al*., 2003; Nosalova *et al*., 2007). Furthermore, we demonstrated a significant decrease in the number of cough efforts evoked by citric acid inhalation in healthy guinea pigs after administration of xanthine derivatives theophylline and theobromine (Mokry *et al*., 2009). Similar results were found after 14 days of ovalbumin sensitization, with significant increases of specific airway resistance after inhalation of saline, citric acid, and histamine. Theophylline and theobromine, however, significantly suppressed the specific airway resistance only in sensitized animals. The *in vitro* evaluation confirmed the beneficial effect of intraperitoneally administered theophylline and theobromine following ovalbumin sensitization. Both xanthine derivatives decreased the *in vitro* airway reactivity compared to the guinea pigs without treatment (Mokry *et al*., 2009).

A significant part of our research was dedicated to the evaluation of antitussive and bronchodilating activities of selective PDE inhibitors (Mokry and Nosalova, 2011). In our experiments with healthy non-sensitized guinea pigs, we demonstrated the antitussive action of vinpocetin, the selective PDE1 inhibitor, cilostazol, the selective PDE3 inhibitor, and of zaprinast, the selective PDE5 inhibitor. Their effect was accompanied by decreased *in vivo* airway reactivity to histamine. In ovalbumin-sensitized animals, a significant antitussive activity was found after administration of vinpocetin, zaprinast and citalopram, the selective PDE4 inhibitor, with simultaneous decrease of *in vivo* airway reactivity to ovalbumin. The *in vivo* response to inhalation of histamine in sensitized animals was influenced only after administration of cilostazol and zaprinast, suggesting participation of PDE3 and PDE5 in inflammatory processes and airway hyperresponsiveness evoked by repeated sensitization by ovalbumin. *In vitro* experiments confirmed beneficial effects of intraperitoneally administered cilostazol and citalopram in both healthy and ovalbumin-sensitized animals. Currently, activity of PDE7 inhibitor BRL 50491 and its combination with rolipram (PDE4 inhibitor) and salbutamol (beta_2_-agonist) is being evaluated. Preliminary results showed significant suppression of inflammation and cough in ovalbumin-sensitized animals.

Concluding these results, we can confirm that in future the selective inhibition of PDE (especially subtypes 3, 4, and 7) as well as xanthine derivatives with their manifold pharmacological properties may assume their position in the therapy of cough and some diseases associated with bronchial hyperresponsiveness. Their anti-inflammatory potential in the therapy of inflammation and airway hyperresponsiveness will be utilized, together with their relaxing effect on smooth muscle.

## Prevention of asthma and polyphenolic substances

Recently increasing attention has been paid to the use of polyphenolic substances in the field of respiratory tract. Polyphenols represent important bioactive molecules in plant foods and possess many biological activities in the airways. They are able to inhibit the release and synthesis of bronchoconstricting mediators and to impair the increased amount of inflammatory cytokines, chemokines, eosinophils and allergen species antibodies during immunoallergic airway inflammation resulting in bronchodilatory and antiinflammatory effects. Furthermore, epidemiological studies contain data supporting the idea that health benefits associated with fruits, vegetables and red wine in the diet are probably linked with polyphenols that most likely reduce the occurrence of asthma symptoms (Varraso *et al*., 2009).

In light of these finfiongs, polyphenols became the object of our interest in experimental research of allergic asthma. Of the large group of these natural substances, products with high concentrations of flavonoids and resveratrol as a polyphenolic compound (Provinol and Flavin7^®^), a flavonol (quercetin) and stilbene (resveratrol) have been used.

Our studies were aimed at the influence of polyphenols on airway hyperreactivity and allergic inflammation in experimental conditions of allergic asthma after their short- or long-term administration. Experiments were realized under *in vitro* and *in vivo* conditions in order to evaluated airway reactivity provoked by bronchoconstrictor mediators. To confirm a probable antiinflammatory effect of polyphenols, these experiments were supplemented with bronchoalveolar lavage and histological examination of the trachea and lung tissues.

In our experiments, the following results were obtained during monitoring the short-term effect of polyphenols. During *in vivo* conditions, a single oral dose of Provinol, Flavin7®, quercetin and resveratrol caused a significant decrease of specific airway conductance. During *in vitro* experiments, polyphenols decreased the amplitude of contraction in response to bronchoconstrictor mediators (Franova *et al*., 2007, Joskova *et al*., 2009).

**Figure 3:**
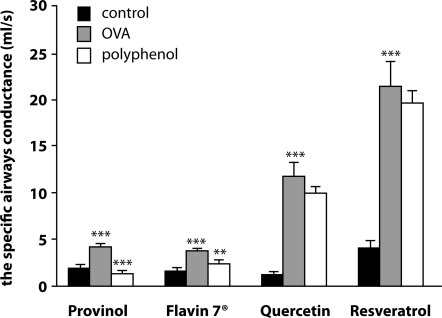
Specific airway conductance of guinea pigs after long-term administration of polyphenols under *in vivo* conditions. Control – control group nonsensitizated guinea pigs without pretreatment, OVA – ovalbumin sensitized guinea pigs without pretreatment, polyphenol – ovalbumin sensitized guinea pigs treated with oral doses of Provinol (20 mg/kg), Flavin7® (2 ml/kg), quercetin (20 mg/kg) or resveratrol (10 mg/kg). Data are expressed as mean±s.e.m., n=10, *p<0.05; **p<0.01; ***p<0.001

After long-term administration, only a mixture of the polyphenolic compounds Provinol and Flavin7 confirmed the ability to reduce airway hyperreactivity during both *in vivo* (Figure 3) and *in vitro* conditions.

The antiinflammatory properties of polyphenols were evaluated after long-term administration using quantification of proinflammatory cytokines IL-4 and IL-5 in bronchoalveolar lavage and eosinophils in the tracheal mucosa and lung tissue. Our results showed antiinflammatory properties of combined polyphenolic compounds in the airways (decrease IL-4, IL-5 levels and eosinophil account), but not of polyphenols alone (Franova *et al*., 2009; 2011; Joskova *et al*., 2011).

In conclusion, we can summarize the most important findings of our experiments. The polyphenolic compounds Provinol and Flavin7^®^ posseses efficient antiasthmatic activity. They cause bronchodilation and also suppress asthmatic inflammation in the airways. Quercetin and resveratrol are able to induce acute bronchodilation without antiinflammatory effects. We thus assume that individual components in polyphenolic compounds can positively influence each other. Our findings are in agreement with some epidemiological studies in which polyphenols caused amelioration of asthma symptoms.

Nowadays, experimental research of allergic asthma continues in our Department of Pharmacology. It is aimed at comparing the effects of polyphenols in the airways and classic treatment of asthma recommended by asthma guidelines.
